# Oculomotor behaviors in youth with an eating disorder: findings from a video-based eye tracking task

**DOI:** 10.1186/s40337-024-01084-y

**Published:** 2024-08-21

**Authors:** Ryan H. Kirkpatrick, Linda Booij, Heidi C. Riek, Jeff Huang, Isabell C. Pitigoi, Donald C. Brien, Brian C. Coe, Jennifer Couturier, Sarosh Khalid-Khan, Douglas P. Munoz

**Affiliations:** 1 Centre for Neuroscience Studies, Queen’s University, Botterell Hall, 18 Stuart St, K7L 3N6 Kingston, ON Canada; 2 School of Medicine, Queen’s University, Kingston, ON Canada; 3 Eating Disorders Continuum & Research Centre, Douglas Mental Health University Institute, Montreal, QC Canada; 4 Department of Psychiatry, McGill University, Montreal, QC Canada; 5 Department of Psychiatry and Behavioural Neurosciences, McMaster University, Hamilton, ON Canada; 6 Department of Psychiatry, Queen’s University, Kingston, ON Canada

**Keywords:** Eating disorder, Saccade, Anorexia nervosa, Bulimia nervosa, Eye blink, Pupil response

## Abstract

**Background:**

The oculomotor circuit spans many cortical and subcortical areas that have been implicated in psychiatric disease. This, combined with previous findings, suggests that eye tracking may be a useful method to investigate eating disorders. Therefore, this study aimed to assess oculomotor behaviors in youth with and without an eating disorder.

**Methods:**

Female youth with and without an eating disorder completed a structured task involving randomly interleaved pro-saccade (toward at a stimulus) and anti-saccade (away from stimulus) trials with video-based eye tracking. Differences in saccades (rapid eye movements between two points), eye blinks and pupil were examined.

**Results:**

Youth with an eating disorder (*n* = 65, *M*_*age*_ = 17.16 ± 3.5 years) were compared to healthy controls (HC; *n* = 65, *M*_*age*_ = 17.88 ± 4.3 years). The eating disorder group was composed of individuals with anorexia nervosa (*n* = 49), bulimia nervosa (*n* = 7) and other specified feeding or eating disorder (*n* = 9). The eating disorder group was further divided into two subgroups: individuals with a restrictive spectrum eating disorder (ED-R; *n* = 43) or a bulimic spectrum eating disorder (ED-BP; *n* = 22). In pro-saccade trials, the eating disorder group made significantly more fixation breaks than HCs (*F*(1,128) = 5.33, *p* = 0.023). The ED-BP group made the most anticipatory pro-saccades, followed by ED-R, then HCs (*F*(2,127) = 3.38, *p* = 0.037). Groups did not differ on rate of correct express or regular latency pro-saccades. In anti-saccade trials, groups only significantly differed on percentage of direction errors corrected (*F*(2, 127) = 4.554, *p* = 0.012). The eating disorder group had a significantly smaller baseline pupil size (*F*(2,127) = 3.60, *p* = 0.030) and slower pro-saccade dilation velocity (*F*(2,127) = 3.30, *p* = 0.040) compared to HCs. The ED-R group had the lowest blink probability during the intertrial interval (ITI), followed by ED-BP, with HCs having the highest ITI blink probability (*F*(2,125) = 3.63, *p* = 0.029).

**Conclusions:**

These results suggest that youth with an eating disorder may have different oculomotor behaviors during a structured eye tracking task. The oculomotor behavioral differences observed in this study presents an important step towards identifying neurobiological and cognitive contributions towards eating disorders.

**Supplementary Information:**

The online version contains supplementary material available at 10.1186/s40337-024-01084-y.

## Abstract (plain language summary)

Video based eye tracking is a promising method for studying differences between individuals with and without a psychiatric disease of interest. While some studies have explored oculomotor behaviors in individuals with an eating disorder, much remains unknown. The present study investigated saccades (fast eye movements between two points), eye blinks and pupil responses between female youth (aged 10–25 years) with and without an eating disorder during a pro-saccade (looking at a point) and anti-saccade (looking away from a point) eye tracking task. Individuals with an eating disorder made more pro-saccade guesses, had a smaller pupil size and blinked less before a trial started. In individuals with a restrictive type eating disorder (e.g., anorexia nervosa restrictive type), pupil responses may have a relationship with emotional dysregulation (poorly regulated emotional responses). Overall, this study represents an important step towards identifying oculomotor behavior differences in individuals with an eating disorder compared to controls.

**Supplementary Information:**

The online version contains supplementary material available at 10.1186/s40337-024-01084-y.

## Introduction

Eating disorders (EDs) often commence in adolescence or early adulthood, and frequently persist throughout adulthood if treatment is not received [1, 2]. Behavioral changes early in treatment predicts ED symptom remission, which highlight the importance of early identification and treatment [3]. One-third of individuals with anorexia nervosa (AN) or bulimia nervosa (BN) have a long-standing ED with a median illness duration of 10 years in AN [4, 5]. Factors that determine who responds to treatment and remains free of relapse are unclear. This uncertainty has prompted a search for biomarkers that are able to distinguish between individuals with and without a diagnosis of interest or between individuals who do and do not respond to a treatment. While the search for psychiatric biomarkers remains ongoing, there are many challenges to its success including low sample sizes, a lack of replication studies and diagnostic crossover [6]. Specifically, within EDs, a lack of known physiological mechanisms challenges this search [7]. Therefore, an important first step toward establishing biomarkers is to identify diagnostic phenotypes to gain a better understanding of the differences between individuals with and without an ED.

One promising method of identifying diagnostic phenotypes is video-based eye tracking. The neural circuitry involved in controlling saccades (rapid eye movements between two points) has been thoroughly investigated and includes critical cortical and subcortical areas implicated in neuropsychiatric diagnoses including the limbic cortex, frontal cortex, anterior cingulate cortex and basal ganglia [8–11]. When examining oculomotor behaviors during an eye tracking task saccade, pupil and blink behaviors can also be assessed. For eye movements, a participant’s behavior when looking directly at a stimulus (fixation behavior), the type of saccades made (e.g., guesses made before the brain has had time to process stimuli location (anticipatory saccades), early saccades (express saccades, triggered immediately following stimulus presentation) and saccades made after the brain has had enough time to process visual stimuli (regular saccades)) and time between stimulus presentation and first saccade (saccadic reaction time) can be compared. For pupil responses, the baseline size of an individual’s pupil as well as the speed their pupil constricts or dilates can be compared. For eye blinks, increases or decreases in the amount of blinking compared to the timing of a task can be considered. While saccades are the most well-studied oculomotor behavior, pupil and blink findings in controls and other neurologic conditions suggest their utility in understanding oculomotor behaviors [12–15]. For instance, blinks have been found to have a reliable pattern of suppression before stimulus appearance in controls completing eye tracking tasks [12] and pupil response velocities have been associated with visual acuity in youth with demyelinating disorders [13].

Recently, the use of oculomotor behaviors to investigate neurological and psychiatric disorders has increased and spans diagnoses such as depression, attention-deficit/hyperactivity disorder (ADHD), obsessive-compulsive disorder (OCD), generalized anxiety disorder (GAD), schizophrenia, bipolar disorder and borderline personality disorder [16–24]. For example, adults with ADHD [20, 23], OCD [21, 22] and GAD [21] have been found to make significantly more anti-saccade direction errors. By examining differences in oculomotor behaviors during complex tasks, variations in sensory, motor, autonomic and cognitive control can be identified.

A systematic review of 31 studies on eye tracking in individuals with EDs found that a large portion of studies used food, body or social stimuli [25]. As such stimuli can be aversive to individuals with an ED, the use of neutral stimuli is warranted [26]. Adults with AN have been found to experience more saccadic intrusions (in the form of square-wave jerks) while focusing on a central fixation point [27] and have higher inhibitory error rates in a memory task [28]. In an interleaved pro-saccade/anti-saccade/no-go task, the same research group found no differences between adults with AN and controls on error rate, but that individuals with AN had a faster pro-saccade reaction time [28]. Individuals with binge-eating disorder have shown increased anti-saccade errors during goal-directed tasks involving food stimuli [29–31]. These findings suggest that individuals with an ED may display overall saccadic reaction time changes and decreased inhibitory control during oculomotor tasks, warranting further transdiagnostic investigation. However, such behaviors have not been studied in a youth population. Similarly, blink and pupil behaviors in individuals with an ED have not been extensively studied to-date [32–34]. Studies in individuals with AN have been conflicting, with one finding a lower blink rate compared to controls and another finding no difference [32, 33] whereas pupil response correlated with emotional arousal [34].

The present study aims to investigate oculomotor behaviors in youth with an ED compared to healthy controls. The primary aim is to identify differences in saccade, pupil and blink behaviors between individuals with an ED and healthy controls. The secondary aims are to identify if oculomotor behaviors differ based on ED behavioral phenotype (restrictive versus binge-purge spectrum) and to investigate the association between oculomotor behaviors and other clinical features measured through questionnaires and medical charts. Based on previous findings [27, 28], it is hypothesized that youth with a restrictive spectrum ED will have no saccadic reaction time difference in anti-saccade trials and a shorter pro-saccade reaction time compared to controls [28]. It is also hypothesized that individuals with a binge/purge ED will have shorter saccadic reaction times, make more anticipatory saccades (guesses) and have lower accuracy in both pro-saccade and anti-saccade trials due to the increased urgency identified in binge/purge spectrum EDs [35]. Given the novelty of the topic, analysis of pupil and blinks in youth with an ED are exploratory in nature.

## Methods

The study was reviewed for ethical compliance by the Health Sciences Research Ethics Board of Queen’s University (HSREB #6040314, #6023982) and the Research Ethics Board at McMaster University (HiREB #7593).

### Participants

To be eligible for inclusion, participants had to meet the following criteria: assigned female at birth, 10–25 years of age, no previous participation in a research study with the Queen’s University Eye Movement Lab, have normal hearing, be fluent in the English language and have normal or corrected to normal vision. The age range was selected as an expanded definition of adolescence has been proposed to be consistent with the continuing biological and sociological development occurring in this extended age range [36]. Since females with EDs are more likely to seek treatment than males [37] and given conflicting evidence regarding the relation between oculomotor behaviors and sex [12, 38–43], it was expected that the study may not be able to recruit a sufficient number of males to reliably allow testing for sex and gender differences. Therefore, this study only included female participants. Sex was determined through a self-report questionnaire.

#### Clinical sample

Patients (*n* = 67) were recruited through the outpatient child and adult ED treatment clinics at Kingston Health Sciences Centre and the Eating Disorder Program at McMaster Children’s Hospital (Hamilton, Canada). Eligible patients required a formal diagnosis of a feeding or ED according to the Diagnostic and Statistical Manual of Mental Disorders, Fifth Edition (DSM-5). They had to be medically stable enough to attend a research session. Clinical information (e.g., diagnosis, body mass index (BMI), medications) was extracted from medical charts. Two individuals with an ED were excluded from data analysis; one for difficulty with eye tracking due to excessive eye makeup, and another who had previously completed the eye tracking tasks through another research study.

#### Healthy controls (HC)

All HCs were recruited from Kingston, Canada through word of mouth, community social media groups and targeted social media advertisements. Upon indicating interest, prospective HCs were asked to self-disclose any previous or ongoing psychiatric diagnoses or taking any psychotropic medications. To confirm eligibility, the Mini-International Neuropsychiatric Interview for Children and Adolescents (MINI-KID; for participants under 18 [44]) or Mini-International Neuropsychiatric Interview (MINI; for participants 18+ [45]) was administered by a trained graduate and medical student (author RHK) to screen for psychiatric diagnoses. When needed, diagnoses were confirmed by a psychiatrist (author SKK) or clinical psychologist (author LB). Of prospective controls that participated in the study (*n* = 85), twenty participants were removed from analysis due to the presence of a MINI of MINI-KID psychiatric diagnosis (*n* = 12), previous participation in another Queen’s Eye Movement Lab study (*n* = 7) or poor-quality eye tracking data (*n* = 1). Each HC’s height and weight were measured during their study visit; some HCs did not consent to being weighed or their weight was not collected due to equipment challenges (*n* = 3). If HC was not weighed, they were not included in any weight-related analyses. A portion (*n* = 29) of the HCs that were recruited for and included within this study have been included in a larger publication on saccades in HCs across the lifespan [42].

### Study procedure

Participants from Kingston (Canada) completed the research study at an Eye Movement Lab location in Kingston Health Sciences Centre. Participants from Hamilton (Canada) completed their research study at McMaster Children’s Hospital. All participants were remunerated with a $50 gift card of their choice for Amazon, Indigo, the local mall or Cineplex.

#### Interleaved pro-saccade and anti-saccade task (IPAST)

**Fig. 1:**
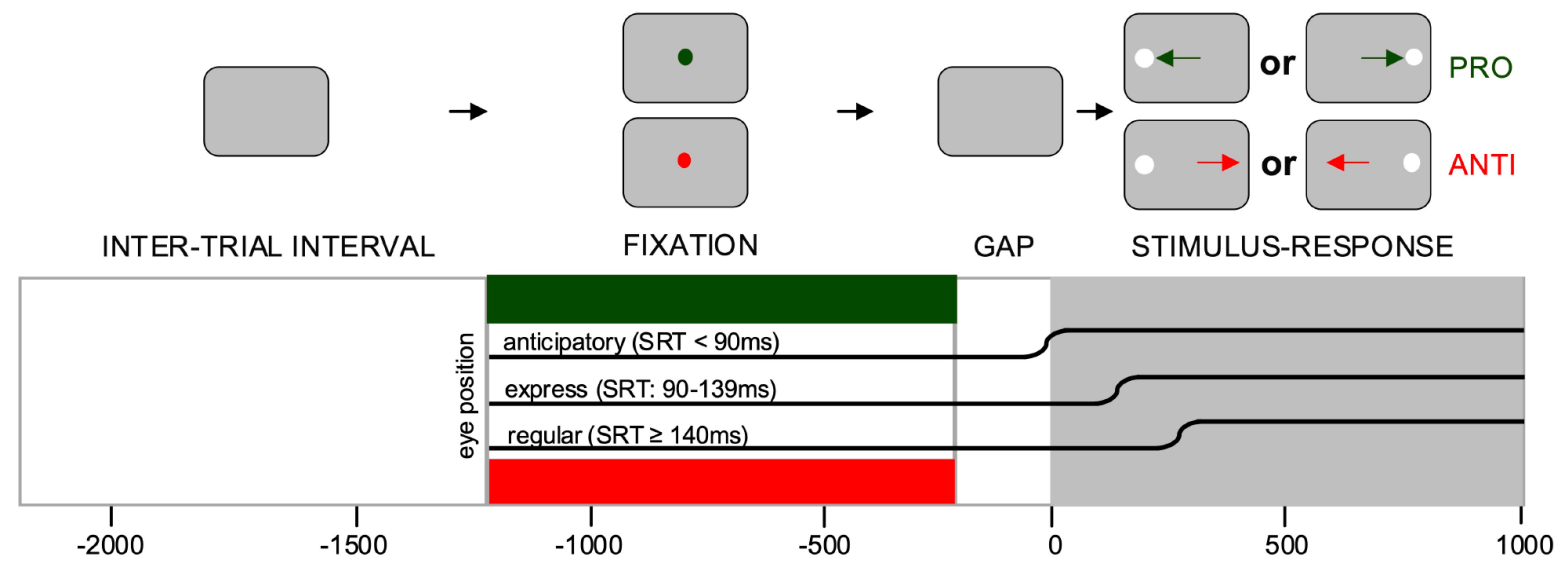
Visual representation of the interleaved pro-saccade/anti-saccade task (IPAST). A green central fixation point indicates a pro-saccade trial (PRO) whereas a red central fixation point indicates an anti-saccade trial (ANTI). Anticipatory saccades are made between 110ms before stimulus presentation and 89ms after stimulus presentation. Express saccades are made between 90ms and 139ms after stimulus presentation. Regular latency saccades are from 140-800ms after stimulus presentation. SRT = saccadic reaction time

IPAST engages the oculomotor circuit by pseudo-randomly alternating between pro-saccade trials that require automatic saccades *toward* a peripheral visual stimulus and anti-saccade trials that require a voluntary saccade *away* from the stimulus. The methods of the IPAST task (Fig. 1) have been extensively described elsewhere [8]. A nine-point calibration and validation procedure was completed before the task and one eye was tracked during the task. In instances of challenging eye tracking, five-point calibration was completed instead (*n* = 2). Mean validation accuracy was 0.53º (*SD* = 0.12º).

Each trial began with the appearance of a central fixation point (FP), displayed for 1000ms, followed by a gap period lasting 200ms with no stimuli on the screen. The color of the central FP (0.5º diameter, 44 cd/m^2^) before the peripheral stimulus appearance served as the signal instructing participants whether to make either a pro-saccade or an anti-saccade after the stimulus appearance. The peripheral stimulus (a white dot) appeared for 1000ms either 10º to the right or left of the central FP. In a pro-saccade trial (green FP), participants were instructed to fixate on the central FP and then look to the peripheral stimulus when it appeared. In an anti-saccade trial (red FP), participants were instructed to look in the “equal but opposite direction” of the peripheral stimulus. Participants were not provided any instruction on blinking or given feedback on the accuracy of anti-saccades. Pro-saccade and anti-saccade trial presentations were randomized with all participants completing 240 trials total. The task was counterbalanced for left versus right peripheral stimuli and pro-saccade versus anti-saccade trials.

#### Questionnaires

Before the COVID-19 pandemic, physical questionnaires were completed by participants (*n* = 41) then transitioned to online collection (*n* = 89) through the secure Qualtrics platform in August 2020.

**Eating disorder inventory third edition (EDI)**. The EDI is a 91-item self-report measure composed of twelve scales and was analyzed to quantify common ED symptomatology and severity. Raw scores were calculated and only the ED risk composite, emotional dysregulation and perfectionism scales were analyzed. Cronbach’s alpha has been reported to range from 0.90 to 0.97 [46].

**Borderline symptom list (BSL)**. The BSL consists of 35 questions on a 5-point scale assessing symptoms of borderline personality disorder [47]. The BSL was included to assess for symptoms of borderline personality disorder given the established comorbidity between binge/purge EDs and borderline personality disorder [48]. Cronbach’s alpha has been previously reported to range from 0.94 to 0.97 [47].

**Barratt impulsivity scale (BIS)**. The BIS is a 30-question measure to quantify impulsiveness and was included as impulsivity has been implicated in anti-saccade performance [49, 50]. Total BIS score was analyzed. Cronbach’s alpha has been previously reported to range from 0.69 to 0.80 [51].

**Suicide behaviors questionnaire-revised (SBQ)**. The SBQ is a 4-item self-report questionnaire measuring suicidal ideation, threat of suicide attempt and the likelihood of a future suicide attempt [52]. The SBQ was included as a measure of suicidal behavior since the prevalence of suicidal behaviors are higher in individuals with an ED compared to the general population and prevalence rates differ between individuals with restrictive versus binge/purge type EDs [53]. Cronbach’s alpha has been previously reported as 0.64 [54].

#### Eye movement recording and apparatus

Eye movements, pupil response and eye blinks were recorded using the EyeLink 1000 Plus video-based eye tracker (SR Research, Ottawa, Canada) at a sampling rate of 500Hz. Participants were seated 60cm away from a 17-inch visual screen with 1280 × 1024 pixel resolution, with their chin resting on a head rest and forehead stabilizer. Tasks were performed in darkness and silence except for the controlled presentation of visual stimuli.

### Data analysis

#### Data processing

All oculomotor variables were processed using the standardized data processing pipeline used by the Queen’s Eye Movement Lab that was described in detail [55] including the saccade, blink and pupil metrics used within the present study (Table 1).

**Table 1:** Oculomotor behaviors

Metric	Definition
**Saccade**	
Fixation break	Saccade away from the fixation point without a return saccade made during the fixation period.
Anticipatory saccade	Saccade made before visual processing between −110-89ms relative to peripheral stimulus onset.
Express latency saccade	Fastest visually triggered saccade (correct or direction error) from 90-139ms relative to peripheral stimulus onset.
Regular latency saccade	Saccade made between 140-800ms relative to peripheral stimulus onset.
Correct pro-saccade	Saccade towards the peripheral stimulus. Can be express or regular latency.
Pro-saccade direction error	Saccade away from the peripheral stimulus. Can be express or regular latency.
Correct anti-saccade	Saccade away from the peripheral stimulus. Can be express or regular latency.
Anti-saccade direction error	Saccade towards the peripheral stimulus. Can be express or regular latency.
Saccadic reaction time	Amount of time (ms) from peripheral stimulus appearance to the first saccade.
Saccade amplitude	Mean amplitude of correct pro-saccades in degrees.
Corrected anti-saccade error	An anti-saccade direction error (towards the peripheral stimulus) followed by a second saccade away from the peripheral stimulus.
**Pupil**	
Baseline pupil size	Median pupil size (pixels) 150-200ms after fixation point onset.
Peak pupil constriction velocity	Maximum rate of constriction (200-1200ms after fixation point onset).
Pupil dilation velocity	Speed of pupil dilation before peripheral stimulus appearance (-50-0ms relative to peripheral stimulus onset).
**Blink**	
Blink probability	Likelihood of eye closure for a given period of time.
- Intertrial interval (ITI) blink probability	Blink probability after the disappearance of the peripheral stimulus of one trial and before the fixation point appearance for the next trial (-2000- -1500ms).
- Fixation (FIX) blink probability	Blink probability during fixation point presentation (-900- -400ms).

Note: Definitions reflect those described in Coe et al. (2024) [55]

#### Saccade metrics

Fixation breaks were instances in which the participants made a saccade (> 2º in amplitude) away from the central FP without returning during the fixation period. Saccadic reaction time (SRT) was the time from peripheral stimulus appearance to the first saccade. Median SRT for all correct trials was calculated for each participant. Each trial with an identifiable behavior was designated as fixation break, anticipatory saccade, express saccade or regular latency saccade with express and regular saccade trials being designated correct or direction error.

Saccades (> 2º in amplitude) initiated from 110ms before stimulus appearance to 89ms after stimulus appearance were defined as anticipatory saccades and were not evaluated as correct or a direction error. Express saccades (> 2º in amplitude) were defined as the fastest visually triggered saccades, with SRTs of 90-139ms [56–58]. Regular saccades (> 2º in amplitude) were saccades with an SRT of 140-800ms.

Trials were scored as correct if the first saccade made after stimulus appearance was in the correct direction as per task instruction. Trials were scored as direction errors if the first saccade made after stimulus appearance was in the wrong direction as per task instruction. Specifically, express and regular pro-saccade direction errors were a saccade in the opposite direction of the peripheral stimulus 90-139ms and 140-800ms after stimulus presentation, respectively. Express and regular anti-saccade direction error were a saccade made towards the peripheral stimulus from 90-139ms and 140-800ms after stimulus presentation, respectively.

The corrected anti-saccade error rate is the proportion of corrected anti-saccade errors divided by the total number of anti-saccade errors irrespective of saccade latency in line with previous studies [59]. Pro-saccade amplitude represents the mean amplitude of correct pro-saccades in degrees towards the peripheral stimulus located 10º from the center of the screen to either side. Anti-saccade amplitudes were not reported since anti-saccade amplitude is extremely variable and therefore their utility is limited. Indeed, some individuals (HCs and EDs) look off the screen, rather than the exact opposite location of the peripheral stimulus.

#### Pupil metrics

Human participants generate a characteristic pupil response in IPAST that consists of an initial constriction response after FP appearance followed by a dilation response that precedes stimulus appearance [60]. Pupil metrics were calculated using previously described methods [43]. Median baseline pupil size was determined separately for pro-saccade and anti-saccade trials and was defined as the average pupil size 150-200ms after central FP appearance. No difference was expected between pro-saccade and anti-saccade baseline pupil size since the calculation reflects a time period before awareness of the trial type occurs. Peak pupil constriction velocity was the maximum rate of constriction after FP appearance. Pupil dilation velocity was calculated immediately prior (-50-0ms) to peripheral stimulus appearance.

#### Blink metrics

Blink probability (the likelihood of eye closure for a given period of time) was calculated using a previously described algorithm (a logical array that identified when the eye was open versus closed separately for each trial and each participant then averaged) [12]. Blink probability was used as it has been shown to be more temporally sensitive than blink rate [12]. Based on previous findings on control behavior during IPAST revealing that blinks are suppressed before stimulus presentation, blink probability was compared within the intertrial interval (ITI; the time between the end of the previous trial and the presentation of the FP for the next trial) and while the FP was presented on the screen (the “FIX” period; Fig. 1). Specifically, blinks between − 2000ms and − 1500ms from stimulus appearance were analyzed for ITI and between − 900ms and − 400ms for FIX.

### Power analysis

Power calculations were based on the findings of Phillipou and colleagues [28], which reported that adults with AN had significantly shorter pro-saccade reaction times compared to HCs, with moderate-large effect size. Such effect size would require 27 individuals with an ED and 27 HCs to obtain a significant effect at the *p =* 0.05 level (two-tailed), with a power of 0.80. Given the number of oculomotor outcome measures and the need to correct for multiple comparisons, a sample of 56 patients and 56 controls was deemed sufficient to test the main hypothesis at the level of *p =* 0.001. Considering the potential for loss of data due to low quality (+/- 5%), a sample of 60 individuals with an ED and 60 controls was deemed sufficient to test the primary hypothesis to obtain the expected effect size (*f* = 0.40), with a power of 0.80, at *p* = 0.001 and inclusion of covariates. Given the subgroup and correlational analyses with demographic and clinical variables were exploratory in nature, their investigation was not reflected in the power analysis calculation.

#### Statistical analyses

Statistical analyses were conducted using SPSS statistics version 29. All analyses were first conducted comparing individuals with an ED to HCs. For secondary exploratory analyses, the ED group was separated into two subgroups based on two common ED presentations: patients with a restrictive spectrum ED (ED-R; *n* = 43, including AN restrictive subtype (AN-R) and other specified feeding or ED, restrictive type (OSFED-R)) and patients with a bulimic spectrum ED (ED-BP; *n* = 22; including AN binge/purge subtype (AN-BP) and bulimia nervosa (BN)). The ED subgroups were created to align with other ED literature differentiating between individuals with a restrictive ED versus binge/purge ED given the identified differences in behaviors, cognition and personality traits between ED presentations [61]. Given the prevalence of diagnostic crossover within EDs [62], it is important to note the ED subgroups only represent each individual’s state at the time of their participation in the study. Tukey’s Honest Significant Difference test was used to determine between group differences when a significant overall effect existed.

Multivariate General Linear Models (GLMs) were conducted for pro-saccade and anti-saccade metrics separately. For saccades and pupil, four separate GLMs were conducted comparing the ED group to HCs and then the two subgroups (ED-R and ED-BP) to HCs for pro-saccade and anti-saccade trials independently. Blink analyses did not have separate analyses for pro-saccade and anti-saccade trials.

**Medication effects on pupil metrics.** Given the established role of psychotropic medications on pupil behavior [63], psychotropic medication usage was added as a covariate to the pupil GLMs.

**Demographic and clinical variables.** For oculomotor variables that were significant in the group analyses (see above), Pearson’s or point-biserial correlations (in the case of psychotropic medication) were conducted to examine the associations with relevant demographic and clinical variables (age, BMI, presence of psychotropic medications, BSL, BIS total score, SBQ and EDI scales).

## Results

### Participants

130 participants were included in the study (*N*_ED_ = 65, *N*_HC_ = 65).

#### ED

Seventeen patients were recruited from McMaster Children’s Hospital and 48 were recruited from Kingston Health Sciences Centre. Two identified as transgender or non-binary. Of those that reported their ethnicity, 86.7% were white (*n* = 52). The diagnostic composition of the group was AN-R (*n* = 38), AN-BP (*n* = 11), BN (*n* = 7) and OSFED (*n* = 9). The highest level of education attended or completed was elementary school (*n* = 15), high school (*n* = 33), college/trade school (*n* = 2), university undergraduate (*n* = 15) and university graduate (*n* = 1). In the ED-R and ED-BP groups, 22 out of 43 and 5 out of 22 were not taking any psychiatric medications, respectively (Supplementary Table 1).

#### HC

Of those that reported their gender (*n* = 60) and ethnicity (*n* = 63), all identified as cisgender and 71.4% (*n* = 45) were white. As per inclusion criteria, no HCs were on psychotropic medications. The highest level of education attended or completed was elementary school (*n* = 17), high school (*n* = 30), college/trade school (*n* = 3), university undergraduate (*n* = 11), university graduate (*n* = 2) and university professional (*n* = 1).

The three subgroups (ED-R, ED-BP, HC) did not significantly differ in age (*F*(2,127) = 2.980, *p* = 0.054), however the groups differed on BMI (*F*(2,124) = 9.540, *p* < 0.001; Table 2). The ED-R group had the lowest BMI (*M*_ED−R _= 19.5, *SD*_ED−R _= 2.6, range = 11.6-25.1) followed by the ED-BP group (*M *_ED−BP _=22.2, *SD*_ED−BP _= 2.7, range = 15.8–36.5) with HCs having the highest BMI (*M*_HC _= 23.2, *SD*_HC _= 5.1, range = 14.8–39.5).

**Table 2:** Demographic characteristics and questionnaire responses between subgroups

	HC (*n* = 65)		ED-R (*n* = 43)		ED-BP (*n* = 22)				
	*M*	*SD*	*M*	*SD*	*M*	*SD*	*F*	*df*	*p*
Age (years)	17.9	4.3	16.4	3.0	18.6	4.0	2.980	2, 127	0.054
BMI	23.2^*,†^	5.1	19.5^*,‡^	2.6	22.2^†,‡^	4.7	9.540	2, 124	< 0.001
BSL	7.6^*,†^	11.3	29.4^*^	23.0	39.5^†^	24.0	33.177	2,127	< 0.001
SBQ	3.8^*,†^	1.7	7.0^*^	5.2	8.3^†^	4.7	16.316	2,126	< 0.001
BIS	59.8	11.4	53.2	26.4	63.0	27.8	2.029	2,125	0.136
EDI-EDRC	117.7^*,†^	18.1	151.4^*,‡^	22.1	178.6^†,‡^	31.1	69.868	2,120	< 0.001
EDI-ED	2.1^*,†^	2.9	6.4^*,‡^	4.8	11.9^†,‡^	5.7	46.494	2, 124	< 0.001
EDI-P	9.4^*,†^	5.1	13.0^*^	6.4	13.0^†^	4.3	7.091	2,126	0.001

^*,†,‡^Each superscript symbol denotes a subset of study groups that significantly differ from another at the 0.05 level. BIS = Barratt Impulsivity Scale, BMI = body mass index, BSL = Borderline Symptom List, ED-BP = eating disorder binge/purge subgroup, ED-R = eating disorder restrictive subgroup, EDI-ED = EDI Emotional Dysregulation Scale, EDI-EDRC = EDI Eating Disorder Risk Composite, EDI-P = EDI Perfectionism Scale, EDI = Eating Disorder Inventory 3, HC = healthy controls, SBQ = Suicide Behaviors Questionnaire

### Questionnaires

The ED-BP group reported the highest level of borderline personality disorder symptoms, suicidal behaviors, impulsivity, ED symptomatology and emotional dysregulation (Table 2). The ED-R and ED-BP reported equal levels of perfectionism while the ED-R group reported the lowest levels of impulsivity.

### Group-level analyses (ED vs. HCs)

Psychotropic medications did not present a significant relationship with saccade (*p* > 0.282) or blink (*p* > 0.341) metrics within the ED group; therefore, it was not included as a covariate in group or subgroup-level analyses. For pro-saccade trials, the ED group made significantly more fixation breaks (*F*(1,128) = 5.325, *p* = 0.023) and significantly more anticipatory pro-saccades (*F*(1,128) = 6.690, *p* = 0.011) than the HC group (Table 3). Groups did not significantly differ on median SRT, correct express pro-saccades, correct regular pro-saccades or mean correct pro-saccade amplitude (Table 3). No significant differences were found on any anti-saccade trial behaviors (fixation breaks, anticipatory saccades, express saccade errors, regular saccade errors, corrected anti-saccade errors or median SRT) when comparing individuals with an ED versus HC (Table 3).

**Table 3:** Saccade parameters by group

	HC (*n* = 65)		ED (*n* = 65)				
	*M*	*SD*	*M*	*SD*	*F*	*df*	*p*
**Pro-saccade**							
Fixation Breaks	5.4	6.1	8.0	6.7	5.325	1, 128	**0.023**
Anticipatory	8.8	7.3	12.4	8.7	6.690	1, 128	**0.011**
Express Correct	31.6	20.1	31.5	15.7	0.000	1, 128	0.992
Regular Correct	50.8	23.4	43.9	22.3	3.001	1, 128	0.086
Median SRT	158	31	150	28	1.969	1, 128	0.163
Mean Amplitude – Correct	9.7	0.5	9.6	2.3	0.002	1, 128	0.962
**Anti-saccade**							
Fixation Breaks	7.3	10.7	10.1	11.1	2.175	1, 128	0.143
Anticipatory	5.3	6.0	7.3	6.1	3.634	1, 128	0.059
Express Error	11.3	9.8	12.8	10.6	0.692	1, 128	0.407
Regular Error	9.8	8.6	8.9	6.8	0.602	1, 128	0.439
Median SRT	235	31	241	40	0.066	1, 128	0.376
Corrected Errors (%)	90.8	11.5	92.6	9.9	0.936	1, 128	0.335

ED = eating disorder group, HC = healthy controls, M = mean, SD = standard deviation, SRT = saccadic reaction time

The ED group had a significantly lower baseline pupil size in both pro-saccade (*F*(1, 128) = 3.599, *p* = 0.030) and anti-saccade (*F*(1, 128) = 3.587, *p* = 0.031) trials. The ED group also had a significantly lower dilation velocity compared to the HC in pro-saccade (*F*(1, 128) = 3.303, *p* = 0.040) but not anti-saccade trials (*F*(1,128) = 2.893, *p* = 0.059). Constriction velocity did not significantly differ between groups during pro-saccade (*F*(1,128) = 2.216, *p* = 0.031) or anti-saccade (*F*(1,128) = 0.801, *p* = 0.451) trials.

The ED group had a significantly lower blink probability during the ITI (*F*(1,128) = 4.781, *p* = 0.031). The blink probability of the ED and HC groups did not significantly differ during the fixation period on pro-saccade (*F*(1,128) = 0.018, *p* = 0.895) or anti-saccade trials (*F*(1,128) = 0.003, *p* = 0.960).

### Subgroup level analyses (ED-R vs. ED-BP vs. HC)

**Fig. 2:**
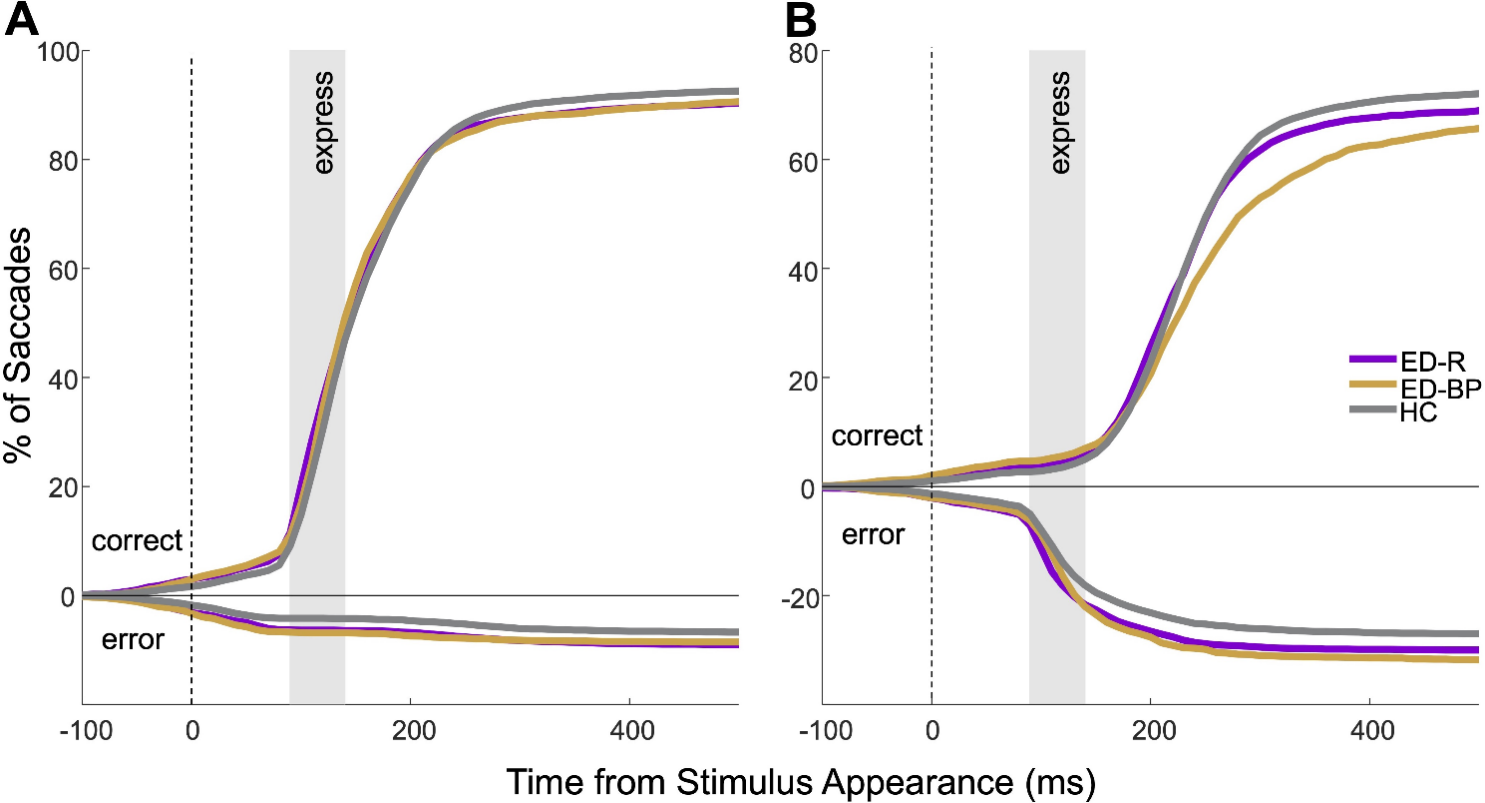
Cumulative distribution of saccades as a function of latency. Traces above the x axis represent the cumulative number of correct trials whereas traces below the x axis represent error trials. ED-BP = eating disorder binge/purge subgroup, ED-R = eating disorder restrictive subgroup, HC = healthy control. **(A)** Pro-saccade cumulative distributions. **(B)** Anti-saccade cumulative distributions

**Fig. 3:**
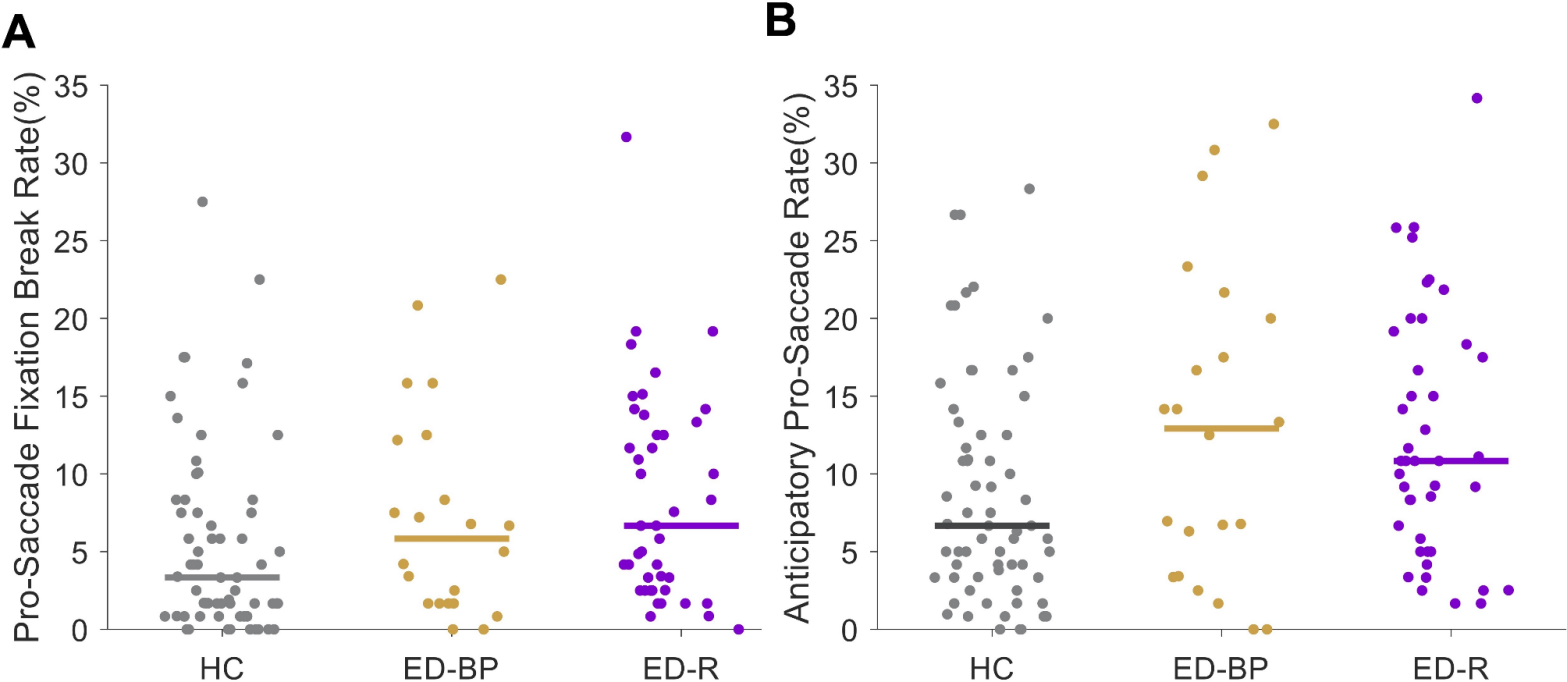
Scatter plots of pro-saccade behaviors across subgroups. Horizontal data lines represent group medians. **(A)** Rate of fixation breaks during pro-saccade trials by subgroup. **(B)** Rate of anticipatory pro-saccades by subgroup. ED-BP = eating disorder binge/purge subgroup, ED-R = eating disorder restrictive subgroup, HC = healthy control

Overall saccade behaviors by subgroup are displayed in Fig. 2. On pro-saccade trials, subgroups did not significantly differ on fixation breaks (Fig. 3A). The ED-BP group made the most anticipatory pro-saccades followed by the ED-R group with the HC group making the least (*F*(2, 127) = 3.375, *p* = 0.037; Fig. 3B). No significant differences were found on median SRT, correct express pro-saccades or correct regular pro-saccades between subgroups (Table 4). Mean correct pro-saccade amplitude did not significantly differ between subgroups with all groups having a mean amplitude of 9.7º (*SD*_ED−R_ = 0.5º, *SD*_ED−BP _= 0.3º, *SD*_HC _= 0.5º). The corrected anti-saccade error rate showed a significant difference across subgroups (*F*(2, 127) = 4.554, *p* = 0.012) such that the ED-R group had the highest percentage of anti-saccade errors corrected (*M* = 95.3%, *SD* = 6.1%) and the ED-BP had the lowest (*M* = 87.5%, *SD* = 13.5%) with HC in the middle (*M* = 90.8%, *SD* = 11.5%). No other anti-saccade behaviors were significantly different across subgroups (Table 4).

**Table 4:** Saccade parameters by subgroup

	HC (*n* = 65)		ED-R (*n* = 43)		ED-BP (*n* = 22)				
	*M*	*SD*	*M*	*SD*	*M*	*SD*	*F*	*df*	*p*
**Pro-saccade**									
Fixation Breaks	5.4	6.1	8.3	6.7	7.2	6.7	2.869	2, 127	0.060
Anticipatory	8.8	7.3	12.2	7.9	12.9	10.1	3.375	2, 127	**0.037**
Express Correct	31.6	20.0	31.9	15.8	30.9	15.8	0.024	2, 127	0.976
Regular Correct	50.8	23.4	43.8	21.2	44.2	24.8	1.491	2, 127	0.229
Median SRT	158	31	149	27	152	31	1.038	2, 127	0.357
Mean Amplitude – Correct	9.7	0.5	9.7	0.5	9.7	0.3	0.013	2, 127	0.987
**Anti-saccade**									
Fixation Breaks	7.3	10.7	9.3	9.3	11.7	14.2	1.432	2, 127	0.243
Anticipatory	5.3	6.0	7.3	5.8	7.3	6.7	1.804	2, 127	0.169
Express Error	11.3	9.8	13.1	10.3	12.2	11.4	0.397	2, 127	0.673
Regular Error	9.8	8.6	8.6	6.7	9.5	7.0	0.404	2, 127	0.669
Median SRT	235	31	235	31	253	50	2.306	2, 127	0.104
Corrected Errors (%)	90.8	11.5	95.3	6.1	87.5	13.5	4.554	2, 127	**0.012**

ED-BP = eating disorder binge/purging subgroup, ED-R = eating disorder group restrictive subgroup, HC = healthy controls, M = mean, SD = standard deviation, SRT = saccadic reaction time

No significant differences were found on pupil metrics when compared across subgroups (ED-R versus ED-BP versus HC; Supplementary Table 2).

The ED-R group had the lowest ITI blink probability, followed by the ED-BP group with the HC group having the highest ITI blink probability (*F*(2, 125) = 3.625, *p* = 0.029). No significant differences were found on blink probability during the fixation period for pro-saccade (*F*(2,127) = 0.021, *p* = 0.979) or anti-saccade (*F*(2,127) = 0.039, *p* = 0.961) trials.

### Correlations with demographic and clinical features

Within the ED group, various significant positive correlations between oculomotor behaviors and demographic and clinical variables were found. Specifically, individuals with an ED who were older had a greater ITI blink probability (*r* = 0.286, *p* = 0.021, 95%CI = 0.045–0.495). Furthermore, use of psychotropic medications was associated with greater pro-saccade baseline pupil size (*r* = 0.320, *p* = 0.009, 95%CI = 0.082–0.523) and with greater anti-saccade baseline pupil size (*r* = 0.317, *p* = 0.010, 95%CI = 0.079–0.520). A lower BMI was associated with a greater corrected anti-saccade error rate (*r *= -0.298, *p* = 0.016, 95%CI = -0.505–0.058). No other significant correlations between demographic and clinical variables of interest with the eye tracking outcome measures were found.

Within the ED-R group, pro-saccade pupil dilation velocity correlated negatively with psychotropic medication use (*r *= -405, *p* = 0.007, 95%CI=-0.619— -0.119), BSL scores (*r *= -0.324, *p* = 0.034, 95%CI = -0.569– -0.027), SBQ scores (*r *= -0.441, *p* = 0.003, 95%CI = -0.654— -0.162), BIS scores (*r *= -0.314, *p* = 0.043, 95%CI = -0.564– -0.011) and EDI emotional dysregulation scale scores (*r *= -0.357, *p* = 0.019, 95%CI = -0.593— -0.063). Baseline pupil size during pro-saccade trials showed a significant positive correlation with psychotropic medications (*r* = 0.311, *p* = 0.042, 95%CI = 0.012–0.559) and BSL scores correlated negatively with pro-saccade fixation breaks (*r *= -0.369, *p* = 0.015, 95%CI = -0.603– -0.078; Supplementary Table 3).

Within the ED-BP group, no significant correlation between any of the clinical and demographic variables and oculomotor variables were identified (Supplementary Table 4).

## Discussion

The primary goal of this study was to determine whether differences exist between EDs and HCs in saccade, pupil and blink behaviors during a structured interleaved pro-saccade and anti-saccade task (IPAST). Overall, differences between individuals with an ED and HCs were identified in pro-saccade behaviors, baseline pupil size and blink probability. This study is the first to extensively study these metrics in youth with an ED and suggests that video-based eye tracking may hold promise as a method to elucidate neurocircuitry changes in female youth with an ED.

Surprisingly, the present study found that pro-saccade trials produced more differences between individuals with an ED and HCs than anti-saccade trials. Specifically, individuals with an ED made more fixation breaks and anticipatory saccades (early guesses about the location of the stimulus) in pro-saccade, but not anti-saccade, trials compared to HCs. Individuals with an ED with binge/purge behaviors (ED-BP) made the most anticipatory pro-saccades, followed by individuals with a restrictive ED (ED-R) with HCs making the least pro-saccade anticipations. While anticipatory saccades have not been previously studies in EDs, an increased number of anticipatory saccades has been noted within numerous psychiatric groups including borderline personality disorder [24, 64], ADHD [20], OCD and schizophrenia and has been suggested to reflect dysfunction between the frontal eye field and the basal ganglia [65]. The findings of increased anticipatory saccades in individuals with various psychiatric diagnoses is important as it is common in many studies to remove before trial (e.g., fixation breaks) and early trial (e.g., anticipatory saccades) behavior from analysis. For example, when exploring the relationship between anxiety and anti-saccade behaviors, anticipatory saccades and behaviors during the fixation period were not considered [66]. Therefore, future research should consider further investigating non-visually triggered saccadic behaviors such as fixation breaks and anticipatory saccades.

This study found that individuals with an ED had a lower baseline pupil size than HCs, but this difference was not significant after separating the ED group by subgroup. Across all individuals with an ED, baseline pupil size was significantly positively correlated with psychotropic medication use, such that psychotropic medication use led to an increased baseline pupil size. Previous studies have noted that individuals taking selective serotonin reuptake inhibitors (SSRIs) had significantly larger pupil diameters [63]. While the subgroup analysis (ED-R versus ED-BP versus HC) was not significant, the median baseline pupil size of the ED-BP group was higher than the HC, which was higher than the ED-R. Given that the majority of the ED-BP group (77.3%) was taking psychotropic medications, medication use may explain the presence of an increased baseline pupil size in the ED-BP group and the significant positive relationship between psychotropic medications and baseline pupil size. However, given that the ED-R group had the lowest pupil size, the significant group level difference may be driven by the ED-R suggesting that ED symptoms (without medication) may correspond to a decreased pupil size. When exploring the associations within the ED-R group, correlations between pro-saccade pupil dilation velocity and numerous measures that capture a facet of emotional dysregulation (BSL, SBQ, BIS and the emotional dysregulation scale of the EDI) were identified. Specifically higher levels of emotional dysregulation were associated with lower pro-saccade pupil dilation velocity. This suggests that pupil responses may be related to emotional dysregulation in individuals with a restrictive spectrum ED.

Finally, individuals with an ED were less likely to blink during the ITI. Previous analysis has demonstrated that blink probability was highest during ITI, representing a release from working memory during a structured task [12]. Blinks may be a measure of engagement such that blinking decreases during times of increased task engagement [67]. While the decreased blink rate in the ED group may suggest their increased engagement, the increased blink probability during the ITI demonstrated by the HC may suggest that HCs more fully understood task timing and constraints and used the ITI as a cognitive release period between trials whereas the ED group did not. It is interesting to note that, despite making fewer blinks during the ITI (which may correspond to increased task attention), individuals with an ED did not make less errors than HCs in pro-saccade or anti-saccade trials.

Unexpectedly, there were overall no significant associations between oculomotor behaviors and clinical features. Aside from the relation between measures of emotional dysregulation and pupil dilation velocity within individuals with a restrictive spectrum ED, the correlations were mostly small and not statistically significant. The lack of a significant association between BMI and oculomotor behaviors is of particular interest as there is some evidence that some other neurobiological or cognitive factors altered in AN revert after weight restoration. For example, in one neuroimaging study, it was found that individuals with AN who were partially weight restored had reduced cortical thickness compared to both individuals with AN who were fully weight restored, and compared to healthy controls [68]. Contrarily, a systematic review of cognitive flexibility in individuals with AN showed that both individuals with acute and recovered AN demonstrate cognitive inflexibility compared to controls [69]. These findings may suggest that the differences identified in oculomotor behaviors of individuals with an ED during IPAST may be less impacted by weight than other neurobiological factors. However, it is important to note that the lack of correlations between eye tracking measures and BMI may also be due to the specific sample of individuals with EDs that participated in the present study. Only 10 individuals in the restrictive subgroup and 3 individuals in the binge/purge ED subgroup had a BMI of less than 18. This is likely, in part, because to participate in the study, individuals with an ED had to be stable enough to attend an off-unit research lab, limiting individuals most medically unwell (and with the lowest BMIs) from participating. Future studies, including a larger sample of individuals who are underweight, are needed to further understand the moderating role of clinical features such as BMI on the association between ED diagnosis and oculomotor behaviors.

While eye tracking methodologies in EDs have been previously employed, the tasks used differ considerably with the specific stimuli, task parameters and participant characteristics varying between studies. For example, studies have employed eye tracking tasks in which food [29] or body stimuli [70] were utilized. This lack of standardization may be a barrier to the determination of biomarkers in psychiatric diagnoses [6]. Therefore, the use of easily reproducible tasks, such as IPAST, is important to not only validate findings within a psychiatric diagnosis, but also across psychiatric diagnoses. To date, pro-saccade and anti-saccade tasks have been conducted in psychiatric diagnoses such as schizophrenia, ADHD, borderline personality disorder, depression and bipolar disorders and have been suggested to be an important tool for their examination [19, 71–73]. However, the use of such tasks has not been used consistently to investigate EDs. This study presents a step towards the use of a standardized task (IPAST) to allow more thorough examination of EDs.

### Limitations

The interpretation and generalizability of this study are limited by the specific population of individuals with an ED that were included as they may not reliably be representative of all individuals with an ED. Specifically, the present study included only females and a low number of gender-diverse individuals, and thus findings may not be generalizable to males and non-cisgender individuals with an ED. Results may also not be reliably applied to community samples with nonclinical levels of disordered eating, or to individuals who are not being referred for treatment to specialized ED programs. Additionally, while the relatively large sample size allowed for comparisons between restrictive and binge/purge spectrum presentations of ED, the present study did not have the statistical power to stratify findings by DSM diagnosis. Moreover, the presence of comorbid diagnoses or psychotropic medication within the ED group may have impacted the findings. However, studying samples without comorbid conditions or medication would not be representative and may limit clinical generalizability. Finally, due to the cross-sectional nature of this study, caution should be taken when interpreting these findings in individuals and settings not reflected in this study.

## Conclusions

This study suggests that differences in oculomotor behaviors may exist between individuals with and without an ED. Individuals with an ED made more fixation breaks and guesses on pro-saccade trials than people without an ED. Further, individuals with an ED had a lower baseline pupil size across both trial types. The specific pattern of oculomotor behaviors in ED may further depend on restrictive versus binge-purge features of the ED, especially before peripheral stimulus appearance. That is, unlike binge-spectrum EDs, restrictive EDs may have a low pupil dilation velocity, which in turn is associated with higher levels of emotional dysregulation. Finally, while individuals with an ED may suppress blinking more than individuals without an ED, this does not appear to translate to an improved performance. Future studies should extend and replicate the findings of the present study by utilizing the IPAST in individuals with an ED, particularly males.

### Electronic supplementary material

Below is the link to the electronic supplementary material.

Supplementary Material 1

## Data Availability

The datasets generated and/or analysed during the current study are not publicly available due privacy concerns but may be available from the corresponding author on reasonable request.
