# Supplementary material for: Oculomotor behaviors in youth with an eating disorder: findings from a video-based eye tracking task
Source: J Eat Disord. 2024 Aug 21;12:121. doi: 10.1186/s40337-024-01084-y (PMC11337776; doi:10.1186/s40337-024-01084-y)
Supplement: Supplementary file 1 — Supplementary Material 1 [file 40337_2024_1084_MOESM1_ESM.docx]

**Supplementary Table 1.** Breakdown of psychotropic medications taken by individuals with an eating disorder at the time of study participation.

|  | ED-R (*n*=43) | ED-BP (*n*=22) |
| --- | --- | --- |
| Medication Classification | *n* (%) | *n* (%) |
| Antidepressant | 15 (34.9)_ | 12 (54.5) |
| - SSRI | 12 (27.9) | 6 (27.3) |
| - SNRI | 3 (7.0) | 5 (22.7) |
| - Other | 1 (2.3) | 2 (4.7) |
| Antipsychotic | 11 (25.6) | 7 (31.8) |
| Benzodiazepine | 2 (4.7) | 2 (4.7) |
| Total (any psychotropic medication) | 21 (48.8) | 17 (77.3) |

ED-BP=eating disorder purging subtype, ED-R=eating disorder group restrictive subtype, SNRI=serotonin and norepinephrine reuptake inhibitor, SSRI=selective serotonin reuptake inhibitor. *Note:* Some individuals may be taking more than one type of psychotropic medication.

**Supplementary Table 2.** Pupil parameters by subgroup.

|  | HC | | | ED-R | | | | | | ED-BP | | | | |  | |  | | | |  |
| --- | --- | --- | --- | --- | --- | --- | --- | --- | --- | --- | --- | --- | --- | --- | --- | --- | --- | --- | --- | --- | --- |
|  | *M* | *SD* | *M* | | | | *SD* | | *M* | | | *SD* | | *F* | | *df* | | | | *p* | |
| Pro-saccade |  |  |  | |  |  | |  | | |  | |  | | | | | |  | | |
| Baseline Pupil Size (pixels) | 3860 | 1426 | 3540 | | | | 1282 | | 3913 | | | 1529 | | 2.424 | | 2, 127 | | | | 0.069 | |
| Constriction Velocity (pixels/ms) | 961.4 | 535.0 | 894.2 | | | | 502.3 | | 895.6 | | | 584.2 | | 1.578 | | 2, 127 | | | | 0.198 | |
| Dilation Velocity (pixels/ms) | 283.9 | 160.7 | 222.1 | | | | 158.0 | | 238.7 | | | 229.9 | | 2.485 | | 2, 127 | | | | 0.066 | |
| Anti-saccade |  |  |  | |  |  | |  | | |  | |  | | | | |  | | | |
| Baseline Pupil Size (pixels) | 3865 | 1423 | 3513 | | | | 1250 | | 3989 | | | 1621.8 | | 2.517 | | 2, 127 | | | | 0.061 | |
| Constriction Velocity (pixels/ms) | 984.1 | 553.0 | 923.4 | | | | 542.7 | | 1041.3 | | | 794.6 | | 0.970 | | 2, 127 | | | | 0.409 | |
| Dilation Velocity (pixels/ms) | 338.1 | 191.4 | 265.8 | | | | 202.6 | | 285.6 | | | 222.3 | | 2.148 | | 2, 127 | | | | 0.098 | |

ED-BP=eating disorder purging subgroup, ED-R=eating disorder restrictive subgroup, HC=healthy controls, M=mean, SD=standard deviation

**Supplementary Table 3.** Correlation analysis between clinical variables, questionnaires and oculomotor behaviours of individuals in the restrictive eating disorder subgroup.

|  |  | PS Fixation Breaks | PS Anticipatory Saccades | AS Recorrected Errors | PS Baseline Pupil Size | PS Dilation Velocity | AS Baseline Pupil Size | ITI Blink Probability |
| --- | --- | --- | --- | --- | --- | --- | --- | --- |
| Age | *r* | -0.214 | -0.128 | 0.239 | -0.079 | -0.200 | -0.058 | .382^*^ |
|  | *p* | 0.168 | 0.414 | 0.123 | 0.612 | 0.199 | 0.710 | 0.011 |
|  | *n* | 43 | 43 | 43 | 43 | 43 | 43 | 43 |
| Psychotropic Meds | *r* | 0.039 | -0.046 | 0.034 | .311^*^ | -.405^**^ | 0.296 | 0.173 |
|  | *p* | 0.802 | 0.772 | 0.830 | 0.042 | 0.007 | 0.054 | 0.268 |
|  | *n* | 43 | 43 | 43 | 43 | 43 | 43 | 43 |
| BMI | *r* | -0.142 | 0.022 | -.318^*^ | -0.154 | -.360^*^ | -0.138 | 0.214 |
|  | *p* | 0.364 | 0.887 | 0.038 | 0.325 | 0.018 | 0.376 | 0.168 |
|  | *n* | 43 | 43 | 43 | 43 | 43 | 43 | 43 |
| BSL | *r* | -.369^*^ | -0.071 | 0.095 | -0.081 | -.324^*^ | -0.086 | 0.002 |
|  | *p* | 0.015 | 0.651 | 0.546 | 0.605 | 0.034 | 0.584 | 0.989 |
|  | *n* | 43 | 43 | 43 | 43 | 43 | 43 | 43 |
| SBQ | *r* | -0.215 | -0.116 | -0.083 | 0.048 | -.441^**^ | 0.051 | 0.057 |
|  | *p* | 0.167 | 0.460 | 0.595 | 0.758 | 0.003 | 0.743 | 0.716 |
|  | *n* | 43 | 43 | 43 | 43 | 43 | 43 | 43 |
| BIS | *r* | -0.177 | -0.093 | -0.131 | -0.233 | -.314^*^ | -0.217 | 0.144 |
|  | *p* | 0.263 | 0.557 | 0.407 | 0.137 | 0.043 | 0.167 | 0.363 |
|  | *n* | 42 | 42 | 42 | 42 | 42 | 42 | 42 |
| EDI-EDRC | *r* | -0.081 | -0.008 | 0.054 | -0.065 | -0.049 | -0.072 | 0.108 |
|  | *p* | 0.626 | 0.961 | 0.743 | 0.693 | 0.766 | 0.662 | 0.513 |
|  | *n* | 39 | 39 | 39 | 39 | 39 | 39 | 39 |
| EDI-ED | *r* | -0.167 | 0.062 | -0.012 | 0.007 | -.357^*^ | 0.010 | -0.134 |
|  | *p* | 0.283 | 0.694 | 0.940 | 0.963 | 0.019 | 0.950 | 0.393 |
|  | *n* | 43 | 43 | 43 | 43 | 43 | 43 | 43 |
| EDI-P | *r* | 0.013 | 0.147 | 0.001 | 0.157 | -0.131 | 0.159 | 0.159 |
|  | *p* | 0.933 | 0.346 | 0.997 | 0.316 | 0.403 | 0.309 | 0.307 |
|  | *n* | 43 | 43 | 43 | 43 | 43 | 43 | 43 |

* Correlation is significant at the 0.05 level. ** Correlation is significant at the 0.01 level. AS=anti-saccade, BIS=Barratt Impulsivity Scale, BMI=body mass index, BSL=Borderline Symptom List, EDI-ED=EDI Emotional Dysregulation Subscale, EDI-EDRC=EDI Eating Disorder Risk Composite, EDI-P=EDI Perfectionism Subscale, EDI=Eating Disorder Inventory 3, PS=pro-saccade, SBQ=Suicide Behaviours Questionnaire

**Supplementary Table 4.** Correlation analysis between clinical variables, questionnaires and oculomotor behaviours of individuals in the a bulimic eating disorder subgroup.

|  |  | PS Fixation Breaks | PS Anticipatory Saccades | AS Recorrected Errors | PS Baseline Pupil Size | PS Dilation Velocity | AS Baseline Pupil Size | ITI Blink Probability |
| --- | --- | --- | --- | --- | --- | --- | --- | --- |
| Age | *r* | -0.170 | 0.125 | 0.180 | -0.256 | 0.138 | -0.286 | 0.089 |
|  | *p* | 0.449 | 0.579 | 0.422 | 0.251 | 0.539 | 0.197 | 0.695 |
|  | *n* | 22 | 22 | 22 | 22 | 22 | 22 | 22 |
| Psychotropic Meds | *r* | 0.035 | 0.299 | -0.058 | 0.283 | 0.045 | 0.286 | -0.368 |
|  | *p* | 0.876 | 0.177 | 0.798 | 0.202 | 0.843 | 0.197 | 0.092 |
|  | *n* | 22 | 22 | 22 | 22 | 22 | 22 | 22 |
| BMI | *r* | -0.326 | -0.148 | -0.124 | 0.056 | 0.158 | -0.009 | 0.071 |
|  | *p* | 0.138 | 0.510 | 0.582 | 0.804 | 0.482 | 0.969 | 0.754 |
|  | *n* | 22 | 22 | 22 | 22 | 22 | 22 | 22 |
| BSL | *r* | 0.067 | 0.205 | -0.185 | 0.040 | 0.332 | 0.074 | -0.169 |
|  | *p* | 0.769 | 0.359 | 0.410 | 0.860 | 0.132 | 0.744 | 0.451 |
|  | *n* | 22 | 22 | 22 | 22 | 22 | 22 | 22 |
| SBQ | *r* | 0.204 | 0.267 | -0.066 | -0.128 | 0.301 | -0.094 | -0.095 |
|  | *p* | 0.362 | 0.229 | 0.770 | 0.572 | 0.173 | 0.676 | 0.673 |
|  | *n* | 22 | 22 | 22 | 22 | 22 | 22 | 22 |
| BIS | *r* | -0.089 | 0.114 | 0.055 | -0.252 | 0.360 | -0.215 | 0.058 |
|  | *p* | 0.694 | 0.612 | 0.809 | 0.259 | 0.100 | 0.336 | 0.798 |
|  | *n* | 22 | 22 | 22 | 22 | 22 | 22 | 22 |
| EDI-EDRC | *r* | -0.264 | 0.048 | -0.049 | -0.094 | 0.111 | -0.099 | -0.204 |
|  | *p* | 0.234 | 0.831 | 0.827 | 0.678 | 0.623 | 0.662 | 0.363 |
|  | *n* | 22 | 22 | 22 | 22 | 22 | 22 | 22 |
| EDI-ED | *r* | 0.042 | 0.072 | 0.205 | 0.330 | 0.156 | 0.386 | 0.059 |
|  | *p* | 0.857 | 0.755 | 0.374 | 0.144 | 0.501 | 0.084 | 0.801 |
|  | *n* | 21 | 21 | 21 | 21 | 21 | 21 | 21 |
| EDI-P | *r* | -0.219 | -0.122 | -0.036 | -0.175 | 0.242 | -0.146 | 0.352 |
|  | *p* | 0.327 | 0.588 | 0.874 | 0.437 | 0.279 | 0.518 | 0.108 |
|  | *n* | 22 | 22 | 22 | 22 | 22 | 22 | 22 |

* Correlation is significant at the 0.05 level. ** Correlation is significant at the 0.01 level. AS=anti-saccade, BIS=Barratt Impulsivity Scale, BMI=body mass index, BSL=Borderline Symptom List, EDI-ED=EDI Emotional Dysregulation Subscale, EDI-EDRC=EDI Eating Disorder Risk Composite, EDI-P=EDI Perfectionism Subscale, EDI=Eating Disorder Inventory 3, PS=pro-saccade, SBQ=Suicide Behaviours Questionnaire
